# Genome Wide Analysis Reveals the Role of VadA in Stress Response, Germination, and Sterigmatocystin Production in *Aspergillus nidulans* Conidia

**DOI:** 10.3390/microorganisms8091319

**Published:** 2020-08-30

**Authors:** Ye-Eun Son, Hee-Soo Park

**Affiliations:** 1 School of Food Science and Biotechnology, Kyungpook National University, Daegu 41566, Korea; thsdpdms0407@naver.com; 2 Department of Integrative Biology, Kyungpook National University, Daegu 41566, Korea

**Keywords:** VadA, conidia, RNA-seq, ultraviolet stress, sterigmatocystin, germination, *Aspergillus nidulans*

## Abstract

In the *Aspergillus* species, conidia are asexual spores that are infectious particles responsible for propagation. Conidia contain various mycotoxins that can have detrimental effects in humans. Previous study demonstrated that VadA is required for fungal development and spore viability in the model fungus *Aspergillus nidulans*. In the present study, *vadA* transcriptomic analysis revealed that VadA affects the mRNA expression of a variety of genes in *A. nidulans* conidia. The genes that were primarily affected in conidia were associated with trehalose biosynthesis, cell-wall integrity, stress response, and secondary metabolism. Genetic changes caused by deletion of *vadA* were related to phenotypes of the *vadA* deletion mutant conidia. The deletion of *vadA* resulted in increased conidial sensitivity against ultraviolet stress and induced germ tube formation in the presence and absence of glucose. In addition, most genes in the secondary metabolism gene clusters of sterigmatocystin, asperfuranone, monodictyphenone, and asperthecin were upregulated in the mutant conidia with *vadA* deletion. The deletion of *vadA* led to an increase in the amount of sterigmatocystin in the conidia, suggesting that VadA is essential for the repression of sterigmatocystin production in conidia. These results suggest that VadA coordinates conidia maturation, stress response, and secondary metabolism in *A. nidulans* conidia.

## 1. Introduction

The *Aspergillus* species produce many spores for propagation under a variety of environmental conditions [1,2]. Most species reproduce asexual spores, known as conidia, which are light and small and can be easily dispersed in the environment [3,4]. These conidia can be the main infectious agent in humans, and can lead to aspergillosis in immunocompromised patients [5], or to allergic bronchopulmonary aspergillosis [6]. Additionally, fungal conidia contain many mycotoxins, including aflatoxins and sterigmatocystin, which can also lead to carcinogenesis [7]. Conidia have a hard cell wall with a protective layer in order to protect against a variety of aggressive environments, such as osmotic, oxidative, temperature, and ultraviolet (UV) stress [8]. During spore wall formation, a myriad of spore-specific regulators alter the composition of polysaccharides in the cell wall, including α-1,3 glucan, β-1,3 glucan, and chitin [9,10,11,12]. Research assessing cell wall composition has primarily focused on *Aspergillus* species, such as *Aspergillus nidulans* and *A. fumigatus* [13,14,15].

*A. nidulans* is the model fungus for asexual development, as it produces asexual specialized structures, called conidiophores, which bear conidia [16,17]. A conidiophore is a multicellular structure consisting of a vesicle, metulae, phialides, and conidia, and the formation of conidiophores is regulated by three key transcription factors, including BrlA, AbaA, and WetA [2,18,19]. Among these transcription factors, WetA is a key regulator that plays a central role in conidia formation during the late phase of asexual development [20,21]. Two velvet regulators, VosA and VelB, are involved in conidial formation, maturation, and dormancy in *Aspergillus* species [22,23]. These three transcription factors regulate the mRNA expression a thousand conidia-specific genes [21,24,25]. A variety of studies have focused on these three transcription factors; however, other spore-specific genes have not been investigated in detail.

*vadA* is one of the VosA/VelB-activated developmental genes that is specifically expressed during formation of conidia in *A. nidulans* [26]. Previous studies have shown that *vadA* is required for the balance between asexual and sexual development in *A. nidulans*. VadA has also been shown to be involved in conidial viability, conidial stress response, β-glucan biosynthesis, and sterigmatocystin production, suggesting that VadA is a multi-functional regulator in *A. nidulans*. Although the roles of VadA have been investigated, detailed regulatory mechanisms of VadA have not been elucidated. In the present study, a transcriptome analysis identified that deletion of *vadA* leads to significant alterations in the mRNA levels of more than 3000 genes in the conidia of *A. nidulans*. Several secondary metabolite gene clusters and genes associated with polysaccharide metabolic processes are affected by the loss of *vadA*, suggesting that VadA is a key regulator of conidia formation in *A. nidulans*.

## 2. Materials and Methods

### 2.1. Strains and Media

Control (THS30, *pyrG89*; *AfupyrG*^+^; *veA*^+^) [24], *vadA* deletion mutant (Δ*vadA*, THS33, *pyrG89*; *pyroA4*; Δ*vadA*::*AfupyrG*^+^; *veA*^+^), and *vadA* complemented (C’ *vadA*, THS34, *pyrG89*; *pyroA*::*vadA*(p)::*vadA*::FLAG3x::*pyroA*; Δ*vadA*::*AfupyrG*^+^; *veA*^+^) strains [26] were grown on solid minimal medium (MM) with 1% glucose (MMG) [27].

### 2.2. RNA-Sequencing (RNA-Seq) Analysis

Fungal conidia were inoculated onto solid MMG and incubated at 37 °C for 2 days. Conidia were collected from the plates after the two days of incubation, washed using ddH_2_O, and filtrated through Miracloth (Calbiochem, San Diego, CA, USA). Conidia were then collected for total RNA extraction. Total RNA was extracted from fungal conidia using Trizol reagent (Invitrogen, Carlsbad, CA, USA) according to the manufacturer’s instructions, with some modifications. RNA samples were treated with DNase I (Promega, Madison, WI, USA) to remove DNA contamination, and then purified using the RNeasy Mini Kit (Qiagen, Germantown, MD, USA). Library preparation and sequencing was performed by Theragen Bio (Suwon, South Korea).

RNA quality was assessed via analysis of the rRNA band integrity using the Agilent RNA 6000 Nano Kit (Agilent Technologies, Santa Clara, CA, USA). Before cDNA library construction, 2 µg of total RNA and magnetic beads with oligos (dT) were used for poly (A) enrichment. The purified mRNA was disrupted into short fragments and double-stranded cDNAs were immediately synthesized. The cDNAs were subjected to end-repair, poly (A) addition, and connected with sequencing adapters using the TruSeq Stranded mRNA Sample Prep Kit (Illumina, San Diego, CA, USA). Suitable fragments were automatically purified using the BluePippin 2% agarose gel cassette (Sage Science, Beverly, MA, USA), and were selected as templates for PCR amplification. The final library sizes and qualities were evaluated electrophoretically using the Agilent High Sensitivity DNA Kit (Agilent Technologies, Santa Clara, CA, USA). Fragments were between 350–450 bp. The library was sequenced using an Illumina HiSeq2500 sequencer (Illumina, San Diego, CA, USA).2.3. RNA-seq data analysis

RNA-seq data analysis and the filtering process were performed as described previously [28]. Filtered reads were mapped to the *A. nidulans* A4 transcriptome [29] using the aligner STAR v.2.3.0e software [30]. Gene expression levels were measured using Cufflinks v2.1.1 [31] with the gene annotation database from the *Aspergillus* Genome Database (AspGD) [29]. For differential expression analysis, gene level count data were generated using the HTSeq-count v0.5.4p3 [32] tool with the option “-m intersection-nonempty” and “-r option considering paired-end sequence.” Based on the read count data that was calculated, differentially expressed genes (DEG) were identified using the R package TCC [33]. Normalization factors were calculated using the iterative DEGES/edgeR method. DEGs were identified based on a q-value threshold less than 0.05.

### 2.3. Gene Ontology Enrichment Analysis

Gene ontology enrichment analyses were carried out using at AspGD [32] and FungiFun [34] database. To characterize the genes identified from the DEG analysis, gene ontology (GO)-based trend tests were performed using the Fisher’s exact test. *p*-values < 0.001 were considered statistically significant.

### 2.4. Data Availability

All RNA-seq data files are available from the NCBI BioProject database (PRJNA646843).

### 2.5. UV Stress Tolerance Test

The UV stress tolerance test was carried out as described previously [35]. Two-day old conidia (about 100 conidia per plate) were spread on MMG plates. The plates were UV -irradiated using a UV crosslinker (Spectrolinke XL-1000 UV crosslinker, Thomas Scientific, Swedesboro, NJ, USA), and the irradiated plates were further incubated at 37 °C for 48 h. The colony numbers were counted and calculated as the survival ratio to that of the untreated control, in triplicates.

### 2.6. Conidial Germination Test

The germination of conidia was measured as described previously [36]. Briefly, conidia (10^7^ per plate) were spread onto solid MM (containing 0.6% (*w*/*v*) sodium nitrate as nitrogen source) with or without glucose, and incubated at 37 °C. The germination rates of the conidia were assessed every hour after inoculation using a Zeiss Lab.A1 microscope (Jena, Germany) equipped with AxioCam 105 and AxioVision (Rel. 4.9) digital imaging software.

### 2.7. Sterigmatocystin Extraction from Conidia and Thin-Layer Chromatography (TLC) Analysis

Sterigmatocystin was extracted from conidia as described previously, with some modifications [28,37]. Conidia were cultured for 5 days and 10^9^ conidia were mixed with 2 mL of CHCl_3_ and 0.5 mm Zirconia/Silica beads (RPI, Mt. Prospect, IL, USA). Conidia were ruptured using a Mini-Beadbeater (BioSpec Products Inc, Bartlesville, OK, USA) and the organic phase was separated by centrifugation and transferred to new vials. After transfer, the organic phase was evaporated and resuspended in 0.1 mL CHCl_3_. Samples were spotted onto a thin-layer chromatography (TLC) silica plate (Kiesel gel 60, 0.25 mm; Merck) and resolved in toluene:ethyl acetate:acetic acid (8:1:1 *v*/*v*). TLC plates were treated with 1% aluminum hydroxide hydrate (Sigma, St. Louis, MO, USA). Images of TLC plates were captured following UV exposure (366 nm). Quantification of sterigmatocystin and aflatoxin spot intensities was calculated using Image J software. Experiments were performed in triplicate.

### 2.8. Real-Time PCR Analysis

Real-time PCR analysis was conducted as described previously [28]. The RNA was extracted as described above. For cDNA synthesis, the GoScript Reverse Transcription System (Promega, Madison, WI, USA) was used. Gene-specific primers (Table S1) and iTaq Universal SYBR Green Supermix (Bio-Rad, Hercules, CA, USA) were used for quantitative real-time PCR. To calculate the expression levels of the target genes, the 2^−∆∆CT^ method was used. β-actin was used as an endogenous control. All experiments were carried out in triplicate.

### 2.9. Statistical Analysis

Statistical differences between the control and mutant strains were evaluated using unpaired Student’s *t*-tests. Data shown represent the mean ± standard deviation.

## 3. Results

### 3.1. VadA Controls Transcript Expression of a Variety of Genes in Conidia

A previous study showed that loss of *vadA* results in reduced spore viability, reduced trehalose content, reduced tolerance to oxidative stress, and increased β (1,3)-glucan levels and sterigmatocystin production in conidia [26]. To further assess the regulatory role of VadA in conidia, transcriptomic analysis of control conidia and conidia with deletion of the *vadA* gene (Δ*vadA*) was performed. As shown Figure S1, deletion of *vadA* led to differential expression of 3678 genes in *A. nidulans* (fold change > 2.0; q-value < 0.05). 1489 genes were downregulated, and 2189 genes were upregulated in the conidia of Δ*vadA* mutants.

**Figure 1:**
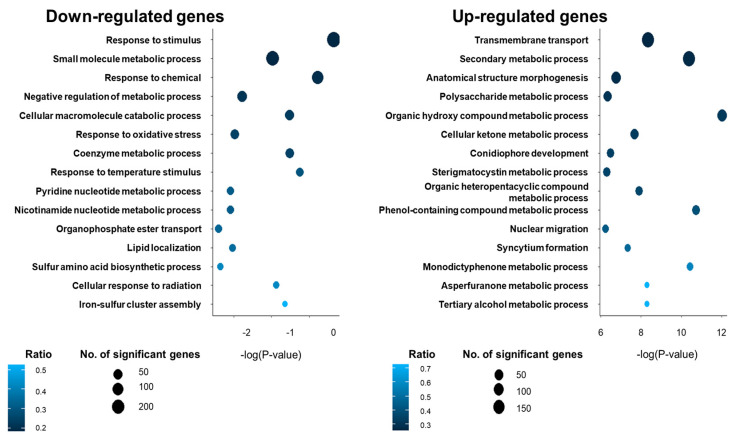
Transcriptomic analyses of Δ*vadA* conidia. GO analysis of DEGs in Δ*vadA* conidia.

To further identify the role of VadA in conidia, gene ontology (GO) analyses were performed [38] using the ASPGD and FungiFun platforms. A large number of genes associated with responses to stimuli, including heat, oxidative, and UV, and small molecule metabolic processes were downregulated in Δ*vadA* conidia (Figure 1). Whereas the genes involved in the transmembrane transport, secondary metabolism, anatomical structure morphogenesis, and polysaccharide metabolic process were upregulated in the Δ*vadA* conidia (Figure 1 and Figure S2). These findings suggest that VadA is involved in polysaccharide metabolic processes, secondary metabolic processes, trehalose formation, and stress response.

### 3.2. VadA Is Required for Expression of Genes Involved in Conidial Wall Integrity

**Figure 2:**
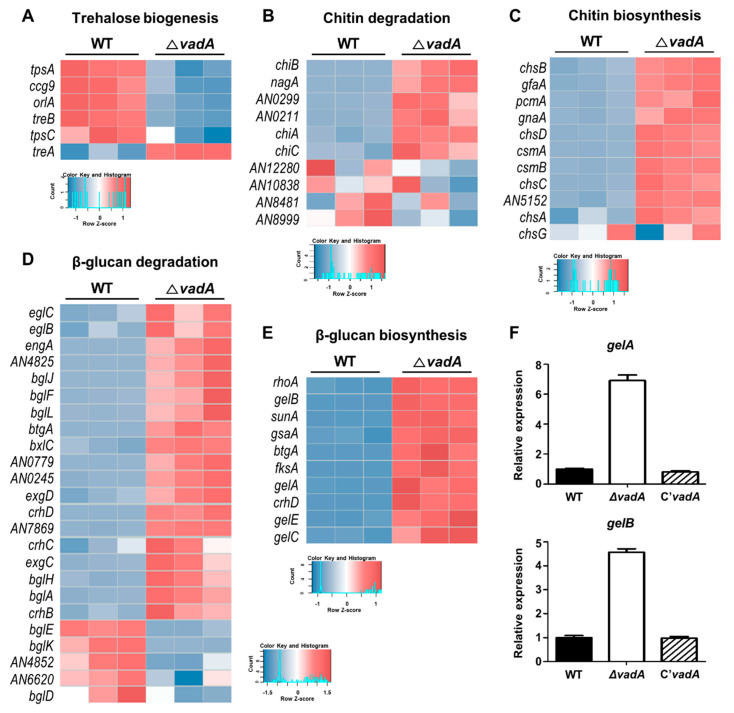
mRNA expression of cell-wall integrity-related genes in Δ*vadA* conidia. (**A**–**E**) Heat map showing the differentially expressed genes (DEGs) of trehalose biogenesis (**A**), chitin degradation (**B**), chitin biosynthesis (**C**), β-glucan degradation and β-glucan biosynthesis (**C**) in control and Δ*vadA* conidia. (**F**) mRNA expression of *gelA* and *gelB* in control (THS30), Δ*vadA* (THS33), and C’*vadA* (THS34) conidia were assessed by qRT-PCR. Error bars indicates the standard errors from the mean of triplicates.

Previous findings have suggested that Δ*vadA* conidia have reduced levels of trehalose, but increased levels of β-glucan, as compared to wild type conidia. VadA was shown to affect the expression of key genes involved in trehalose (*tpsA* and *orlA*) and β-glucan (*fksA*) biosynthesis [26]. In the present study, RNA-seq data revealed that the mRNA expression of a variety of genes associated with trehalose, β-glucan, and chitin biogenesis were affected by deletion of *vadA* (Figure 2). The expression of genes related to trehalose synthesis, such as *tpsA*, *orlA*, *ccg9*, and *tpsC*, were decreased, and the mRNA expression of *treA* (encoding alpha, alpha-trehalase) was increased in the Δ*vadA* conidia (Figure 2A). Interestingly, the mRNA levels of many genes associated with chitin and β-glucan biosynthesis or degradation were increased in the mutant conidia (Figure 2B–F). These data suggest that VadA is required for the gene expression of genes involved in cell wall integrity, as the deletion of *vadA* results in alterations in the content of these carbohydrates.

### 3.3. VadA Is Required for Conidial Germination

**Figure 3:**
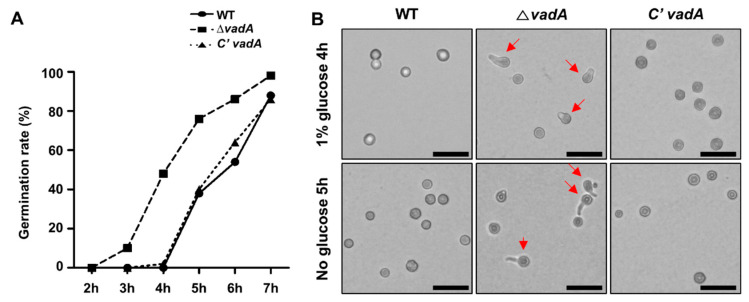
Role of *vadA* in germination. (**A**) Conidial germination rate in control (THS30), Δ*vadA* (THS33), and C’ *vadA* (THS34) conidia inoculated on solid minimal medium with glucose. (**B**) Control (THS30), Δ*vadA* (THS33), and C’ *vadA* (THS34) conidia were inoculated on solid MM (1% glucose), with or without a glucose source and incubated for 4 or 5 h, respectively (bar = 25 µm). Arrows indicate germ tubes.

During spore germination and hyphal growth, the dynamic gene expression for the genes involved in the biosynthesis of chitin when β-glucan biosynthesis is occurred [39,40]. As we observed alterations in the gene expression of genes involved in these pathways in the Δ*vadA* conidia, we hypothesized that *vadA* deletion may affect conidial germination. To test this hypothesis, the conidial germination rate was assessed. As shown Figure 3, the germination rate of the Δ*vadA* conidia was faster than that of the control conidia and complemented strains. The Δ*vadA* conidia, but not the control or complemented conidia, formed germ tubes, suggesting that VadA is required for proper conidial germination.

### 3.4. Deletion of vadA Affects for UV Stress Response

**Figure 4:**
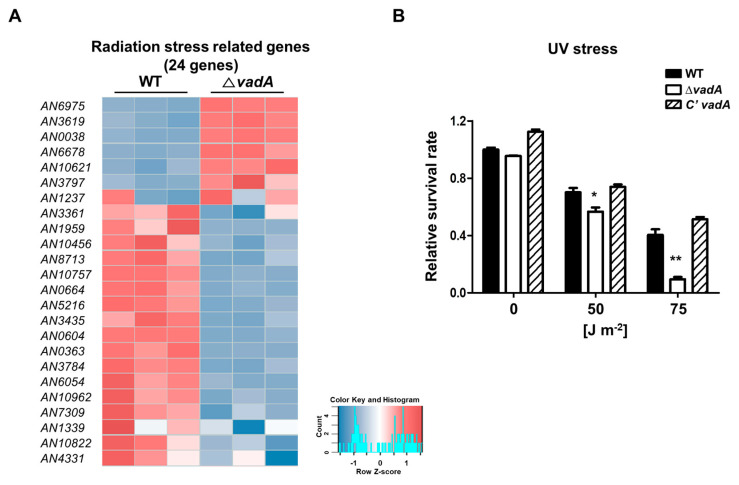
Role of *vadA* in the UV-mediated stress response. (**A**) Heat map showing the DEGs with UV stress response. (**B**) The tolerance of control (THS30), Δ*vadA* (THS33), and C’ *vadA* (THS34) conidia against UV irradiation. About 10^2^ conidia were irradiated with 50 or 75 J/m^2^ of UV at 260 nm (triplicate measurements). Error bars represent the standard deviation (differences between the *vadA* mutants and control strains). ** *p* < 0.01; * *p* < 0.05.

Previous studies have also shown that Δ*vadA* conidia are highly susceptible to oxidative stress [26]. In the present study, functional analysis showed that the expression of stress response-related genes was reduced in the Δ*vadA* conidia (Figure 1). Importantly, the mRNA levels of genes associated with response to radiation were decreased (Figure 4A). Therefore, we assessed the tolerance of the Δ*vadA* conidia against UV stress. As shown Figure 4B, the Δ*vadA* conidia were more sensitive to UV stress than the control conidia. Taken together, these results, which are consistent with previous findings, indicate that VadA is important for UV and oxidative stress response in *A. nidulans* conidia.

### 3.5. Role of VadA in Secondary Metabolite Gene Clusters in A. nidulans Conidia

**Figure 5:**
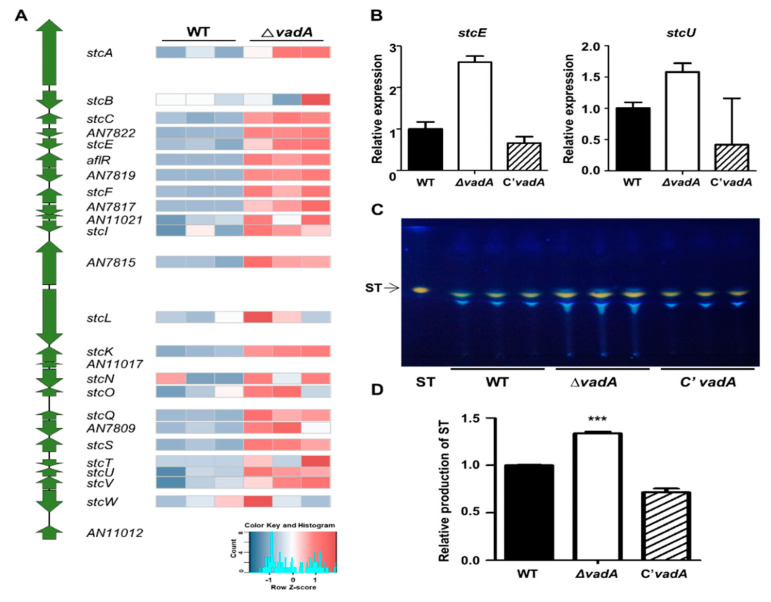
The role of VadA in sterigmatocystin production in conidia. (**A**) Heat map diagram of genes in sterigmatocystin gene clusters. (**B**) mRNA expression of *stcE* and *stcU* in control (THS30), Δ*vadA* (THS33), and C’*vadA* (THS34) conidia were assessed by qRT-PCR. (**C**) TLC image of sterigmatocystin from control (THS30), Δ*vadA* (THS33), and C’ *vadA* (THS34) conidia. (**D**) Relative production of sterigmatocystin shown in (**C**). Error bars represent standard deviation (differences between the *vadA* mutants and control strains. *** *p* < 0.001).

**Figure 6:**
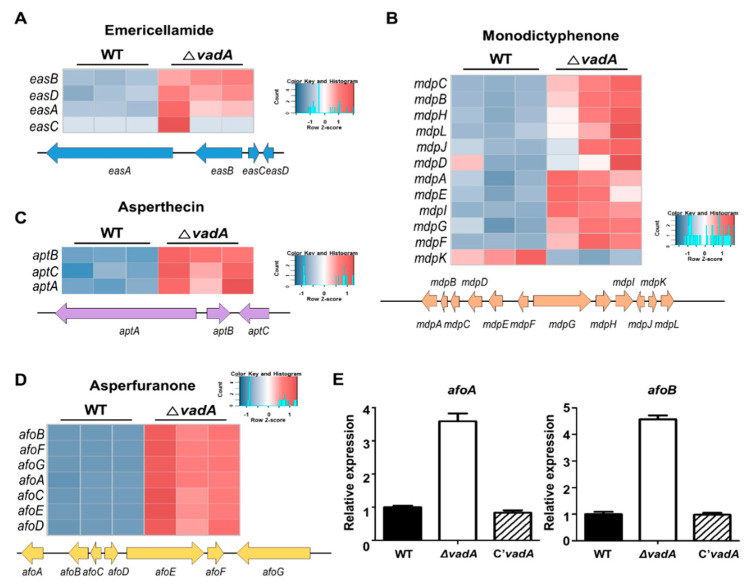
Expression of secondary metabolite gene clusters in Δ*vadA* conidia. (**A**–**D**) Heat map diagram of genes related to emericellamide (**A**), monodictyphenone (**B**), asperthecin (**C**), and asperfuranone (**D**) gene clusters. (**E**) mRNA expression of *afoA* and *afoB* in control (THS30), Δ*vadA* (THS33), and C’*vadA* (THS34) conidia were assessed by qRT-PCR.

Our previous study identified that VadA is required for proper sterigmatocystin production during development [26]. In the present study, we used RNA-seq data to obtain further insight into *vadA* regulation of secondary metabolism in *A. nidulans* conidia. Among the 25 secondary metabolite gene clusters, the mRNA expression of genes related to six secondary metabolites gene clusters was upregulated in the Δ*vadA* conidia (Table 1). In the sterigmatocystin gene cluster, the mRNA levels of a majority of the genes were increased in the mutant conidia (Figure 5A,B). In addition, the amount of sterigmatocystin in the Δ*vadA* conidia was increased as compared to that of control conidia, suggesting that VadA is negatively associated with sterigmatocystin production in conidia (Figure 5C,D). In addition to sterigmatocystin biosynthesis, other secondary metabolites gene clusters involved in the production of asperfuranone, monodictyphenone, emericellamide, and asperthecin were also upregulated with the deletion of *vadA* (Figure 6). Overall, these results indicate that VadA is important for proper secondary metabolism in *A. nidulans* conidia.

## 4. Discussion

Conidia production is tightly regulated by spore-specific transcription factors. In particular, WetA, VosA, and VelB have been identified as key transcription factors in *Aspergillus* species [21,24]. Previous studies identified a variety of target genes of the VosA-VelB complex in conidia in *A. nidulans* [24,25]. VadA is one VosA/VelB-activated developmental gene that is a conidial specific gene in the *Aspergillus* species [26]. Deletion of *vadA* affects the balance between asexual and sexual development, conidial viability, conidial trehalose and β-glucan content, and sterigmatocystin production, suggesting that VadA is a multifunctional protein involved in fungal development and conidial maturation in *A. nidulans*.

To further assess the mode of action of VadA in *A. nidulans*, RNA-sequencing was performed in asexual spores from wildtype and *vadA* deletion mutants. Our analyses revealed that the absence of *vadA* affects the expression of more than 30% of the genes in the *A. nidulans* genome. The genetic changes caused by the deletion of *vadA* are consistent with previous phenotypic findings [26]. The major phenotypes observed in the Δ*vadA* conidia were loss of conidial viability, reduced trehalose content in conidia, and increased conidial sensitivity against UV and oxidative stresses. Our transcriptome analysis indicated that loss of *vadA* led to decreased mRNA expression of genes associated with spore viability (*vosA*, *velB*) [36,41], oxidative stress response (*atfA*, *catA*, *trxA*, *msrA*, *napA*), and UV stress response (*uvsD*, *uvsF*) [42] indicating that VadA is a key regulator of stress response in tolerance in conidia.

Another feature of Δ*vadA* conidia is that they have higher sterigmatocystin content than control conidia (Figure 5). Among the 23 genes in the sterigmatocystin gene cluster, the mRNA expression of 21 genes (nine genes were DEG) were increased in the Δ*vadA* strains. This suggests that upregulation of these genes may affect sterigmatocystin production. In addition, transcripts of genes in gene clusters involved in the production of asperfuranone, asperthecin, emericellamide, and monodictyphenone were also increased in the Δ*vadA* conidia. Several studies have aimed to identify the gene clusters of these secondary metabolites; however, the function of these metabolites in fungal growth and development has not yet been studied. Therefore, more research is needed to elucidate the functional association of *vadA* with these pathways.

Chitin and β-glucan are the primary polysaccharides involved in cell wall integrity in most fungi [9,43]. There are dynamic levels of chitin and β-glucan biosynthesis during hyphal tip extension [44,45]. In conidia, the VosA-VelB complex negatively regulates the expression of various genes, including cell wall-related genes, resulting in the repression of chitin and β-glucan biosynthesis [24]. In the Δ*vadA* conidia, the β-glucan content and the expression of genes involved in β-glucan biosynthesis were increased, suggesting that VadA is also necessary for proper repression of β-glucan biosynthesis in conidia. One possible explanation for the role of VadA in β-glucan biosynthesis in conidia is that VadA indirectly regulates β-glucan biosynthesis through the VosA-VelB complex. Deletion of *vadA* results in decreased mRNA expression of *vosA* and *velB*, two key repressors of β-glucan biosynthesis. However, as the molecular function of VadA has not been studied in detail, further work is necessary to elucidate the molecular roles of VadA.

The new finding in this study is that deletion of *vadA* led to increased germination rates (Figure 3). Several factors are involved in the gemmation, one of the important components is trehalose [40]. This is because trehalose acts as not only a protectant against oxidative stress in spores, but also an energy source during spore germination [46]. A previous study found that the Δ*vadA* conidia contain less amount of trehalose than WT conidia [26] The reduced amount of trehalose in the Δ*vadA* conidia may induce spore germination. Another speculation about the acceleration of the conidial germination rate of the Δ*vadA* conidia is trehalose hydrolysis. A recent metabolomic study proposes that trehalose hydrolysis plays an important role in conidia dormancy breakage and germination [47]. RNA-seq results showed that mRNA expression of *treA* [48], was increased in the Δ*vadA* conidia, speculating that trehalose hydrolysis can be induced, due to the deletion of *vadA*. Further study is needed to clarify the role of VadA in conidial germination.

**Figure 7:**
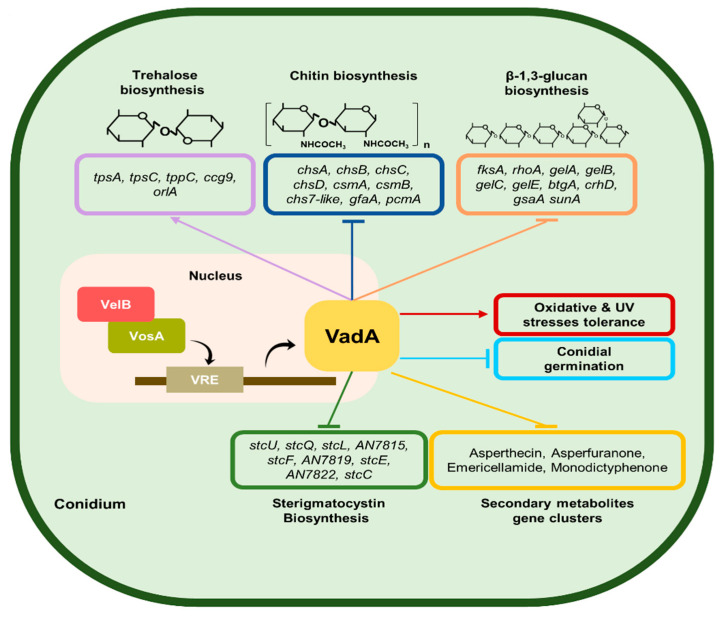
Genetic model for VadA-mediated regulation in *A. nidulans* conidia. A proposed model for the VadA-mediated conidiogenesis is presented.

Based on the previous phenotypic findings and the genome-wide data obtained in the present study, we propose a model of VadA-related regulation in *A. nidulans* conidia (Figure 7). In conidia, VadA primarily affects a variety of biological processes. First, VadA is required for the regulation of mRNA expression of secondary metabolites gene clusters, such as sterigmatocystin, asperfuranone, asperthecin, emericellamide, and monodictyphenone. In particular, VadA may play a role in repressing the expression of sterigmatocystin related genes and the production of sterigmatocystin. Second, VadA represses the mRNA expression of genes associated with cell-wall integrity, thereby affecting the production of polysaccharides, such as β-glucan and chitin. Third, VadA involves in the regulation of mRNA expression of genes associated with trehalose biosynthesis, which then affects oxidative stress response and conidial germination. Forth, VadA is involved in the oxidative and UV stress response in conidia. Overall, these findings suggest that VadA is a conidia-specific regulator that governs metabolic processes and conidial maturation in *A. nidulans*. This study provides insights about how to regulate conidia viability and mycotoxin metabolism in conidia in other pathogenic fungi, such as *A. fumigatus* and *A. flavus*.

## Figures and Tables

**Table 1:** Upregulated genes in secondary metabolite genes clusters in the Δ*vadA* conidia.

Secondary Metabolite Gene Cluster (Number of Genes in the Cluster)	Upregulated Genes in the Δ*vadA* Conidia
Asperfuranone (7)	*afoA*, *afoB*, *afoC*, *afoD*, *afoE*, *afoF*, *afoG*
Asperthecin (3)	*aptA*, *aptB*, *aptC*
Aspyridone (8)	*apdB*, *apdA*, *apdF*, *apdG*
Austinol cluster 1(10)	*ausH*, *ausI*, *ausJ*, *ausK*
Austinol cluster 2 (4)	*ausC*, *ausA*, *ausD*
Derivative of Benzaldehyde1 and F9775 hybrid cluster 1 (9)	*dbaA*, *dbaB*, *dbaC*, *dbaG*, *dbaH*, *dbaI*
Derivative of Benzaldehyde1 and F9775 hybrid cluster 2 (3)	*orsA*, *orsB*, *orsC*
Emericellamide cluster (4)	*easA*, *easB*, *easC*, *easD*
*inp* cluster (6)	*inpC*, *inpD*, *scpR*, *inpE*
*ivo* cluster (2)	*_*
Microperfuranone cluster (3)	*AN3394*, *AN3395*, *micA*
Monodictyphenone cluster (12)	*mdpA*, *mdpB*, *mdpC*, *mdpD*, *mdpE*, *mdpF*, *mdpG*, *mdpH*, *mdpI*, *mdpJ*, *mdpK*, *mdpL*
Penicillin cluster (3)	*acvA*, *ipnA*, *aatA*
Nidulanin A cluster (2)	*AN11080*
Sterigmatocystin cluster (23)	*stcW*, *stcV*, *stcU*, *stcS*, *AN7809*, *stcQ*, *stcO*, *stcN*, *stcL*, *AN7815*, *stcI*, *AN7817*, *stcF*, *AN7819*, *aflR*, *stcE*, *AN7822*, *stcC*, *stcA*, *stcK*, *AN11021*
Terriquinone cluster (4)	*tdiA*, *tdiC*
*pkb* cluster (8)	*cicC*, *cicE*, *pkbA*, *AN6449*, *AN6450*, *AN6451*
*pkdA* cluster (11)	*pkdA*, *AN0524*, *AN0525*, *AN0526*, *AN0527*, *AN0528*, *AN0529*, *AN0530*, *AN0531*, *AN0533*
*pkf* cluster (6)	*AN3225*, *pkfA*, *pkfC*, *pkfD*, *pkfE*, *pkfF*
*pkg* cluster (8)	*AN7072*, *AN7074*, *AN7075*, *AN10884*
*pkh* cluster (8)	*AN2031*, *AN2033*, *AN2034*, *pkhB*, *AN2036*, *AN2037*, *AN2038*
*pki* cluster (8)	*AN3379*, *pkiB*, *pkiC*, *salA*, *AN3383*, *AN3385*, *AN3386*
*xptA*-containing cluster (10)	*xptA*, *AN6785*, *AN6786*, *AN6789*, *AN6791*
*xptB*-containing cluster (4)	*AN7999*, *xptB*, *AN9467*
*sidC* cluster (4)	*AN0606*, *sidC*, *AN0608*

## Supplementary Materials

The following are available online at https://www.mdpi.com/2076-2607/8/9/1319/s1. Figure S1: Heat map showing the clustering of the differentially expressed genes (DEGs) between control and Δ*vadA* conidia (fold change > 2.0 and q-value < 0.05) is presented. Figure S2: Functional enrichment analysis in Δ*vadA* conidia. The charts show categories that were significantly enriched for down-regulated (**A**) or up-regulated (**B**) genes. The analysis was performed using the FungiFun database. Table S1: Oligonucleotides used in this study.
