# Supplementary material for: Genome Wide Analysis Reveals the Role of VadA in Stress Response, Germination, and Sterigmatocystin Production in Aspergillus nidulans Conidia
Source: Microorganisms. 2020 Aug 30;8(9):1319. doi: 10.3390/microorganisms8091319 (PMC7565415; doi:10.3390/microorganisms8091319)
Supplement: Supplementary file 1 [file microorganisms-08-01319-s001.pdf]

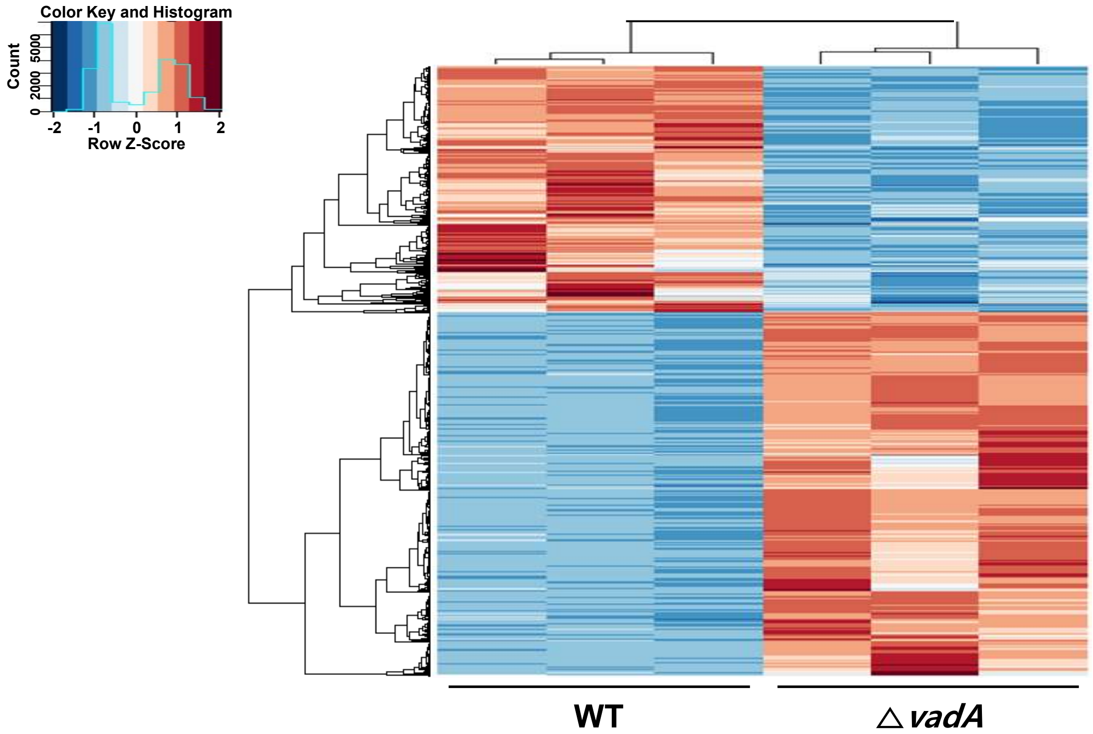

Total: 3,678 genes
(fold change > 2.0 and p-value < 0.5)

A

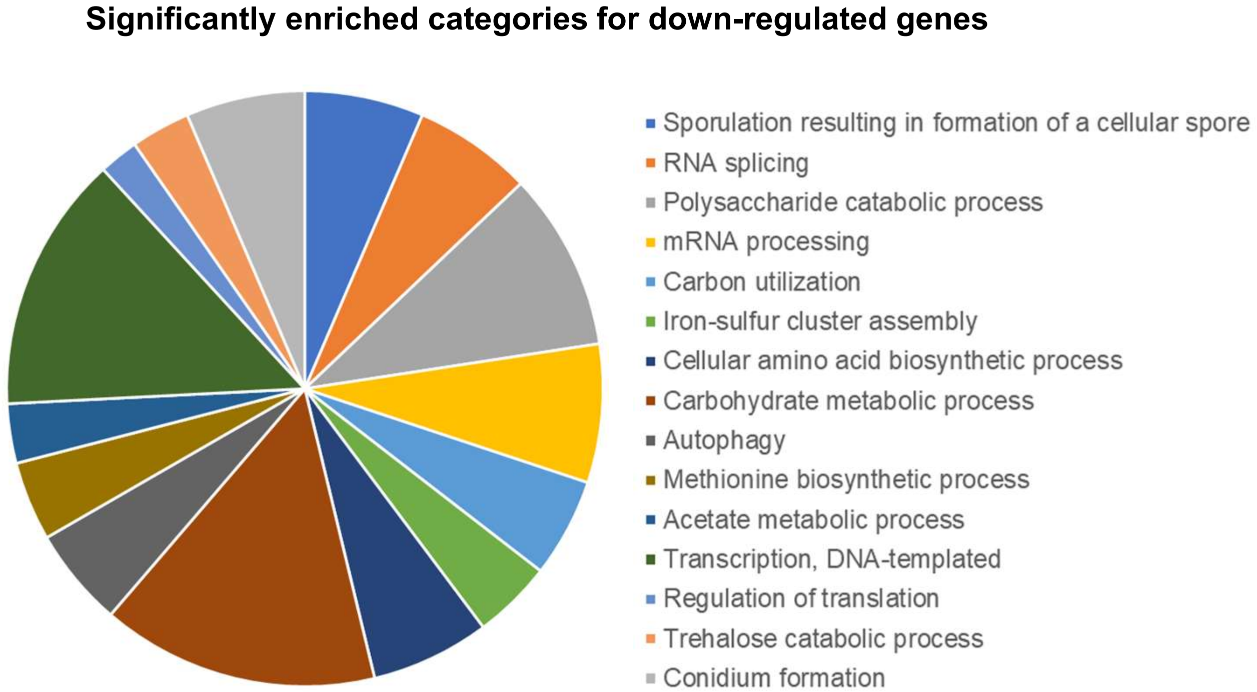
Significantly enriched categories for down-regulated genes
Sporulation resulting in formation of a cellular spore
RNA splicing
Polysaccharide catabolic process
mRNA processing
Carbon utilization
Iron-sulfur cluster assembly
Cellular amino acid biosynthetic process
Carbohydrate metabolic process
Autophagy
Methionine biosynthetic process
Acetate metabolic process
Transcription, DNA-templated
Regulation of translation
Trehalose catabolic process
Conidium formation

B

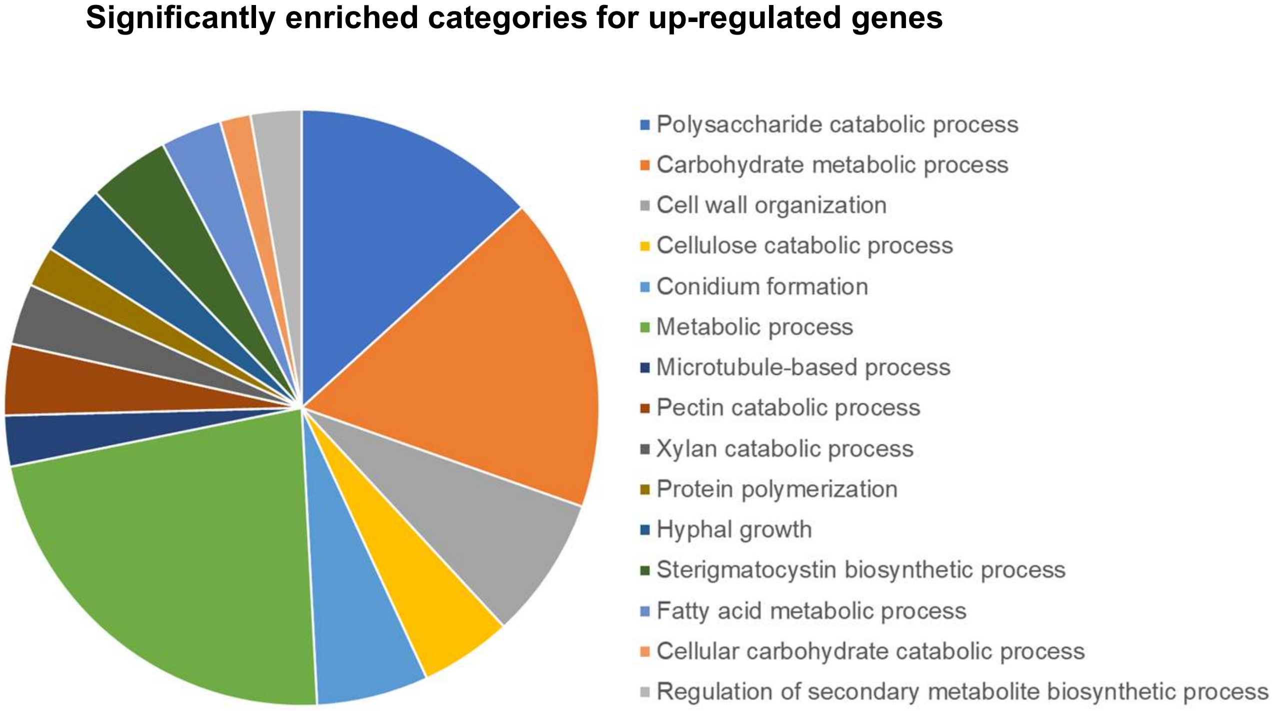
Significantly enriched categories for up-regulated genes
Polysaccharide catabolic process
Carbohydrate metabolic process
Cell wall organization
Cellulose catabolic process
Conidium formation
Metabolic process
Microtubule-based process
Pectin catabolic process
Xylan catabolic process
Protein polymerization
Hyphal growth
Sterigmatocystin biosynthetic process
Fatty acid metabolic process
Cellular carbohydrate catabolic process
Regulation of secondary metabolite biosynthetic process

Table S1. Oligonucleotides used in this study.

Name	Sequence (5′ → 3′)[a]	Purpose
OHS0044	GTAAGGATCTGTACGGCAAC	5' Actin_RT_F
OHS0045	AGATCCACATCTGTTGGAAG	3' Actin_RT_R
OHS0602	CGCATCATCCTCACAAGTTC	5' *stcU* RT_F
OHS0603	TGACCGTGATCTTCTTGTCG	3' *stcU* RT_R
OHS0604	GCTACTGTTCCAGGCGACTA	5' *stcE* RT_F
OHS0605	CACAGCTCTCCATCTCGGTA	3' *stcE* RT_R
OHS0797	ATGACTGGCGTCTACTCTGG	5' *gelA*_RT_F
OHS0798	GGGTTGGAGGTCTTGGAGAA	3' *gelA*_RT_R
OHS1445	CCTGGTGTCCAGGACTTGAT	5' *gelB*_RT_F
OHS1446	CCGGTAATTTCCTTGCCGTT	3' *gelB*_RT_R
OHS1447	ACTACCCTAACCGACCCTCT	5' *afoA*_RT_F
OHS1448	TACATGAGTCTCGCCTCGTC	3' *afoA*_RT_R
OHS1449	GGTCATGTACTTGGGCATCG	5' *afoB*_RT_F
OHS1450	GGAACAGTCTCCTTCTCCGT	3' *afoB*_RT_R
